# Ixekizumab for genetically confirmed type 5 pityriasis rubra pilaris with a CARD14 p.Met119Thr variant^؆^

**DOI:** 10.1016/j.abd.2026.501439

**Published:** 2026-08-06

**Authors:** Omer Faruk Tolun, Yusuf Demir, Burhan Engin

**Affiliations:** Department of Dermatology and Venereology, Cerrahpaşa Faculty of Medicine, Istanbul University-Cerrahpaşa, Istanbul, Turkey

Dear Editor,

The management of Pityriasis Rubra Pilaris (PRP) remains challenging, particularly in patients with genetically confirmed disease refractory to conventional therapies. Although the classification system proposed by Griffiths identifies six subtypes (Table 1), the rarity of familial forms ‒ including type 5 PRP associated with CARD14 mutations ‒ has limited evidence-based treatment recommendations.^1^, ^2^ CARD14 mutations result in constitutive activation of nuclear factor-κB signaling with subsequent upregulation of Interleukin-17A (IL-17A), providing a mechanistic rationale for IL-17A inhibitors in this population.

**Table 1:** Griffiths classification of pityriasis rubra pilaris. Types I–IV and VI are sporadic. Type V is the only familial form, inherited in an autosomal dominant pattern due to gain-of-function CARD14 mutations.

Type	Nomenclature	Age of Onset	Clinical Features	Course	Genetic Basis
I	Classic adult	5^th^–6^th^ decade	Cephalocaudal spread; follicular papules; islands of sparing; PPK	80% resolve within 3yrs	Sporadic
II	Atypical adult	Adulthood	Ichthyosiform features; alopecia; eczematous changes	Chronic; ∼20yrs	Sporadic
III	Classic juvenile	First 2yrs	Similar to type I; generalized	Self-limiting; 1–2yrs	Sporadic
IV	Circumscribed juvenile	Prepubertal	Localized to elbows and knees	Chronic	Sporadic
**V**	**Atypical juvenile (familial)**	**Early childhood**	**Follicular hyperkeratosis; scleroderma-like changes; erythroderma; PPK**	**Chronic; lifelong**	**AD; CARD14 mutations**
VI	HIV-associated	Variable	Nodulocystic acne; lichen spinulosus-like	Chronic	Associated with HIV

PPK, Palmoplantar Keratoderma; AD, Autosomal Dominant. Type V (highlighted) represents the familial form described in the present case.

A 35-year-old woman presented with a lifelong history of widespread erythematous and scaly eruptions, with affected first-degree relatives. Physical examination revealed generalized erythematous, hyperkeratotic, follicular papules and plaques involving the trunk and extremities, with prominent palmoplantar keratoderma (Fig. 1); the baseline Physician Global Assessment (PGA) score was 4 (severe). Whole-exome sequencing identified a heterozygous missense variant in CARD14 (NM_001366385.1: c.356T>C; p.Met119Thr), classified as likely pathogenic per American College of Medical Genetics and Genomics criteria (PM1, PM2, PM5, PP5, BP4), establishing the diagnosis of type 5 PRP. Histopathological examination revealed psoriasiform dermatitis with alternating orthokeratosis and parakeratosis (checkerboard pattern) and a superficial perivascular lymphocytic infiltrate.

**Fig. 1:**
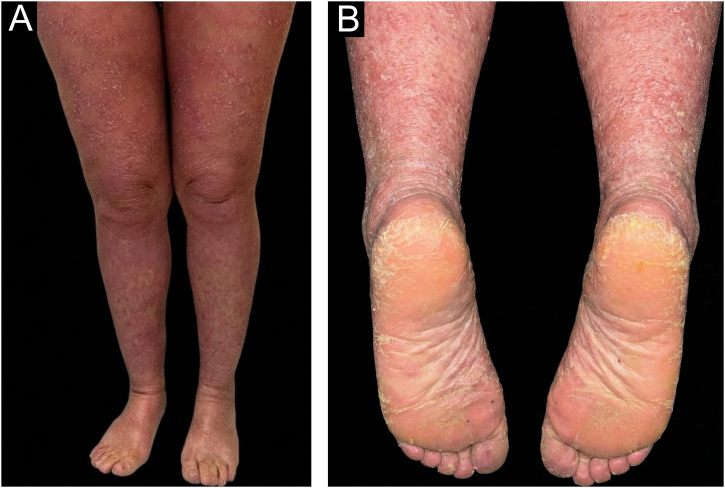
Pre-treatment clinical photographs of a 35-year-old woman with genetically confirmed type 5 pityriasis rubra pilaris harboring a CARD14 p.Met119Thr variant. (A) Anterior view of the lower extremities demonstrating widespread erythematous plaques with characteristic follicular papules. (B) Plantar surfaces demonstrating prominent keratoderma.

Prior topical therapies, including high-potency corticosteroids and salicylic acid, had been ineffective. A 10-year course of acitretin (0.5–0.75 mg/kg/day) yielded only partial response. Given the patient’s desire for pregnancy, acitretin was discontinued owing to its teratogenic potential; methotrexate was similarly contraindicated. Based on the pathophysiological role of IL-17A in CARD14-mutated PRP and emerging evidence supporting IL-17A inhibition (Table 2), ixekizumab was initiated. Ixekizumab was preferred over secukinumab because its single loading dose offers practical advantages over secukinumab’s four-weekly induction injections ‒ a consideration also highlighted in a recent pediatric PRP case.^3^, ^4^

**Table 2:** Reported biologic therapy outcomes in genetically confirmed CARD14-associated pityriasis rubra pilaris.^3^, ^5^, ^6^, ^8^ Summary of published cases of CARD14-mutated PRP or CARD14-Associated Papulosquamous Eruption (CAPE) treated with biologic agents, including the present case.

Study	Patient	CARD14 Variant	Diagnosis	Biologic	Response	Follow-up
Lwin et al.^5^	49 F + 20 M (mother-son)	p.Met119Arg (c.356T>G)	Type V PRP	Ustekinumab 45 mg q12w	Significant improvement within days of first dose; sustained	Sustained on maintenance
McCarthy et al.^8^	Adult F	p.Met119Thr (c.356T>C)	Type V PRP	Ustekinumab	Dramatic improvement	Sustained response
Niedźwiedź et al.^6^	11M	p.Ile123Phe (c.394A>T)	CAPE	Adalimumab → Ustekinumab	Good response to ustekinumab after adalimumab failure	Maintained on ustekinumab
Ouyang et al.^3^	Adult	Novel variant	CAPE	Ixekizumab	Successful treatment of severe phenotype	Sustained response
**Present case**	**35F**	**p.Met119Thr (c.356T>C)**	**Type V PRP**	**Ixekizumab 160/80 mg**	**PGA 4→1 in 2 weeks; pruritus and QoL improvement**	**Remission at 24 weeks; PGA 0–1**

F, Female; M, Male; PRP, Pityriasis Rubra Pilaris; CAPE, CARD14-Associated Papulosquamous Eruption; PGA, Physician Global Assessment; QoL, Quality of Life; q12w, Every 12-weeks.

Ixekizumab was administered per standard psoriasis dosing: 160 mg loading dose at week 0, followed by 80 mg at weeks-2, -4, -6, -8, -10, and -12, with maintenance dosing of 80 mg every 4-weeks. Within 2-weeks, the PGA score improved from 4 to 1 (almost clear) (Fig. 2), with substantial improvement in pruritus and quality of life. At 24-weeks, clinical remission persisted (PGA 0–1) without adverse events. A gradual dose de-escalation with extended dosing intervals is planned.

**Fig. 2:**
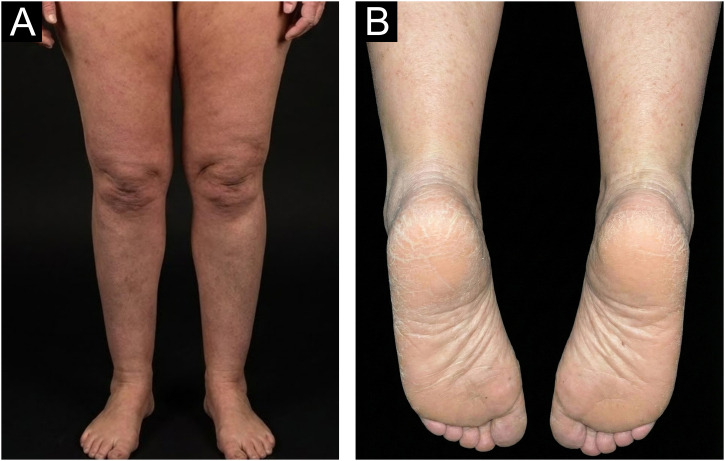
Clinical photographs two weeks after initiation of ixekizumab therapy. (A) Anterior view of the lower extremities showing marked improvement with near-complete resolution of erythema and scaling. (B) Plantar surfaces with significant improvement in keratoderma.

The p.Met119Thr variant resides within the caspase recruitment domain of CARD14, critical for NF-κB activation. Biologic therapies targeting the IL-23/Th17 axis have shown consistent efficacy in genetically confirmed CARD14-associated PRP (Table 2).^3^, ^5^, ^6^, ^7^, ^8^ Lwin et al. reported a mother-son pair with a p.Met119Arg variant who achieved significant improvement within days of ustekinumab initiation.^5^ More recently, Niedźwiedź et al. described an 11-year-old boy with a novel p.Ile123Phe variant and CARD14-associated papulosquamous eruption who responded to ustekinumab after adalimumab failure.^6^ Ferreirinha et al. reported an 8-year-old girl with type IV PRP who achieved near-complete remission by 5-months of ixekizumab after failure of methotrexate.^4^ A systematic review reported 35.7% complete resolution with ixekizumab in non-genotyped PRP patients^7^; in the only prospective ixekizumab trial, none of the 12 participants harbored CARD14 variants, which may partially explain the heterogeneous responses observed.^9^

The p.Met119Thr variant was previously reported in a type 5 PRP patient who responded to ustekinumab.^8^ Ustekinumab has also demonstrated efficacy in acquired PRP; Vieira Granja et al. described a 69-year-old woman with COVID-19 vaccine–triggered PRP who achieved almost complete clearance within 4-weeks of ustekinumab despite CARD14-negative status.^10^ Our case represents the first report of ixekizumab efficacy with this specific variant, suggesting that patients with confirmed CARD14 mutations may be particularly suitable candidates for IL-17A-targeted therapy.

Ixekizumab may be an effective option for genetically confirmed type 5 PRP, especially when conventional therapies are contraindicated. Its shorter half-life compared with acitretin is advantageous for women of childbearing potential. The rapid and sustained response underscores the value of genetic testing in guiding personalized PRP treatment.

## Patient consent

Written informed consent was obtained from the patient for publication of this case and accompanying images.

## Data availability statement

Data sharing is not applicable to this article as no new data were created or analyzed.

## Financial support

None declared.

## Authors’ contributions

Omer Faruk Tolun: The study concept and design; data collection, analysis and interpretation; writing of the manuscript; critical review of the literature; final approval of the final version of the manuscript.

Yusuf Demir: The study concept and design; data collection, analysis and interpretation; writing of the manuscript or critical review of important intellectual content; ıntellectual participation in the propaedeutic and/or therapeutic conduct of the studied cases; critical review of the literature; final approval of the final version of the manuscript.

Burhan Engin: Effective participation in the research guidance; ıntellectual participation in the propaedeutic and/or therapeutic conduct of the studied cases; critical review of important intellectual content; final approval of the final version of the manuscript.

## Research data availability

Does not apply.

## Conflicts of interest

None declared.

## ORCID ID

Omer Faruk Tolun: 0009-0008-3749-2658

Yusuf Demir: 0000-0003-3307-5917

Burhan Engin: 0000-0002-5140-1926
